# Neuroenhancements in the Military: A Mixed-Method Pilot Study on Attitudes of Staff Officers to Ethics and Rules

**DOI:** 10.1007/s12152-022-09490-2

**Published:** 2022-02-28

**Authors:** Sebastian Sattler, Edward Jacobs, Ilina Singh, David Whetham, Imre Bárd, Jonathan Moreno, Gian Galeazzi, Agnes Allansdottir

**Affiliations:** 1 Institute of Sociology and Social Psychology, University of Cologne, Cologne, Germany; 2 Pragmatic Health Ethics Research Unit, Institut de Recherches Cliniques de Montréal, Montréal, Germany; 3 Faculty of Sociology, Bielefeld University, Universitaetsstrasse 25, 33615 Bielefeld, Germany; 4 Department of Psychiatry, University of Oxford, Oxford, UK; 5 Wellcome Centre for Ethics and Humanities, University of Oxford, Oxford, UK; 6 Defence Studies Department, King’s College London, London, UK; 7 Department of Methodology, London School of Economics and Political Science, London, UK; 8 Department of Medical Ethics and Health Policy, University of Pennsylvania, Philadelphia, USA; 9 Department of Biomedical, Metabolic and Neural Sciences, Center for Neuroscience and Neurotechnology (CfNN), University of Modena and Reggio Emilia, Modena, Italy; 10 Dipartimento Di Salute Mentale E Dipendenze Patologiche, Azienda USL-IRCCS Di Reggio Emilia, Reggio Emilia, Italy; 11 Università di Siena, 53100 Siena, Italy

**Keywords:** Neuroenhancement, Human Performance Augmentation, Military, Enhancement Pills, Neural Implants, Neuroprostheses, Ethics

## Abstract

Utilising science and technology to maximize human performance is often an essential feature of military activity. This can often be focused on mission success rather than just the welfare of the individuals involved. This tension has the potential to threaten the autonomy of soldiers and military physicians around the taking or administering of enhancement neurotechnologies (e.g., pills, neural implants, and neuroprostheses). The *Hybrid Framework* was proposed by academic researchers working in the U.S. context and comprises “rules” for military neuroenhancement (e.g., ensuring transparency and maintaining dignity of the warfighter). Integrating traditional bioethical perspectives with the unique requirements of the military environment, it has been referenced by military/government agencies tasked with writing official ethical frameworks. Our two-part investigation explored the ethical dimensions of military neuroenhancements with military officers – those most likely to be making decisions in this area in the future. In three workshops, structured around the *Hybrid Framework*, we explored what they thought about the ethical issues of enhancement neurotechnologies. From these findings, we conducted a survey (*N* = 332) to probe the extent of rule endorsement. Results show high levels of endorsement for a warfighter’s decision-making autonomy, but lower support for the view that enhanced warfighters would pose a danger to society after service. By examining the endorsement of concrete decision-making guidelines, we provide an overview of how military officers might, in practice, resolve tensions between competing values or higher-level principles. Our results suggest that the military context demands a recontextualisation of the relationship between military and civilian ethics.

**Supplementary Information:**

The online version contains supplementary material available at 10.1007/s12152-022-09490-2.

## Introduction

The science fiction inspired image of the biotechnologically enhanced soldier has permeated popular culture throughout the latter half of the 20^th^ and on into the twenty-first century. Recent technological developments demonstrate that some of those capabilities are either already here, or are on the brink of radically transforming the way that we interact with the world (for example: [1–3]). Over the past decade the notion of using emerging technologies to enhance human performance, and in particular cognitive, affective and sensory capacities has attracted a great deal of academic attention. Beyond scholarly interest in the subject, several projects in foresight, technology assessment, and bioethics have addressed the social, legal and ethical aspects of neuroenhancement [e.g., 4, 5, 6, 7, 8, 9]. The majority of discussions around neuroenhancement have tended to focus upon the distinction between therapeutic and enhancing interventions, alongside questions concerning the proper scope of medicine, while some have linked this to broader questions relating to the societal implications of embracing such technology in sports and in the workplace [e.g., 10, 11, 12]. Interest has also emerged in studying the potential of neuroenhancement in and for specific groups, such as medical doctors, scientists, airline pilots or military personnel whose professional duties can greatly impact upon the welfare of others [13–17]. In these contexts, enhancement may be seen as an intervention that allows individuals to better fulfil their professional roles. A recent trinational survey about public attitudes found that the use of brain-computer interfaces in the context of military, police, and security, for example, to monitor fatigue or prevent fear in soldiers and to carry out lie detection/interrogation caused high levels of public concern [18].

Utilising science and technology in order to maximize human performance is a recognized, and often essential, feature of armed forces’ operations. The military is often at the cutting edge of research, contributing significant resources to research and development, making it a highly likely source of neuroenhancement technologies, with various research programs seeking either to *maintain* peak performance in the face of environmental and operational stressors, or to *amplify* performance beyond existing capacities [19]. News reports, open access documents by the United States Defence Advanced Research Projects Agency (DARPA), as well as official military websites allow glimpses into the variety of neuroscience-based research projects that aim at enhancing human performance and capacities [20]. At the same time, in order to operate successfully, the military needs to maintain a high level of secrecy, which greatly distinguishes it from most other sectors of society. Furthermore, the superordinate aim of neuroenhancement in the military is usually not the welfare of the enhanced individual but rather the successful execution of missions and the accomplishment of military objectives. As several scholars have pointed out, this can lead to tensions around the autonomy and consent of soldiers and military physicians when it comes to taking or administering enhancements [21–23].

Resources and activities created for military purposes also have implications for society at large, given the bi-directional flows of technologies, innovations and personnel between civilian and public contexts [24]. These flows have sometimes had transformative effects [25]; e.g., the emergence of the Internet from the military-funded ARPANET and related projects [26]. However, despite the flow of technologies from the military towards civilian applications, there is little societal insight into those military innovations and the degree of oversight that takes place in their development is often unclear. Recognising this, a DARPA-sponsored committee of the US National Academies recommended that all national security agencies engaging in emerging technologies create a mechanism for continuous review of ethical, legal and social issues [22]. This was echoed in a late-2019 report by the Biotechnologies for Health and Human Performance Council of the Department of Defense, which explored the feasibility, applications as well as related ethical, legal, and social aspects (ELSI) of human/machine fusion technologies by the year 2050 [27]. Current legal, security, and ethical frameworks were deemed inadequate and the authors advised the Department of Defence to support the development of forward-leaning policies that minimize the risks and maximize the benefits of human enhancement for the US and its allies. While calling for the scientific and engineering communities to move cautiously, the report also advocated for a ‘whole-of-nation’ approach to cyborg technologies to ensure coordinated federal and commercial investment in this area to avoid being outpaced by other nations.

The first major national ethics framework to be published in October 2017 was the Canadian Defence Research and Development’s “Identifying Ethical Issues of Human Enhancement Technologies in the Military” [28]. It took another four years for the first European code to be published [29] with the French Ministry of the Armed Forces Ethics Committee’s “Reflection on the Augmented Soldier” in September 2020 [30].^1^ While the UK has yet to publish its own ethics framework, work is being undertaken in this area by the Defence Science and Technology Laboratory and other government agencies, as well as high level bilateral conversations with key allies. Noting the global nature of human enhancement technologies is not only a cause for rivalry but also a challenge for allied nations to ensure interoperability, the UK has also been heavily involved with the US-led Multinational Capability Development Campaign (MCDC), designed to collaboratively develop and assess concepts and capabilities to address the challenges associated with conducting joint, multinational and coalition operations between allied NATO forces [32]. While also the US has yet to publish its own national framework, the driving concern of the MCDC report, published in March 2021, is the standardization of definitions and frameworks across NATO allied forces for the sake of ensuring interoperability and operational success of allied armed forces. It identifies a number of ethically salient factors that must be attended to in the ethical development and eventual deployment of Human Performance Augmentation (their preferred term covering this area, including neuro enhancements) in the military. While there will no doubt be much reflection on whether the NATO states have arrived at the right principles, individually or collectively, it is clear that all of the emerging codes and frameworks so far owe a great deal to the hybrid framework first proposed by Lin, Mehlman, and Abney in 2013 [22], either being very similar in terms of principles, scope and purpose, and/or citing the work extensively.

Lin et al.had proposed a framework in the U.S. context comprising a set of “rules” for human enhancement in the military. The authors analysed international humanitarian law, the laws of armed conflict, and US domestic law relevant to the use of enhancements, as well as the medical, research and public health models of bioethical inquiry. In Lin et al*.*’s assessment, bioethics provides a “natural frame” and entry point to analyse human enhancement in the military. However, the subordination of war fighters’ individual welfare to collective mission success proves difficult to align with bioethics’ commitment to reducing harm and promoting individual autonomy. In addition, the authors argue that risk analysis is too quantitative and misses the ethical dimensions of targeting mind, brain and body for optimisation for military purposes. Lin et al*.* propose a “Hybrid Framework” that integrates more traditional bioethical perspectives while trying to take into account the unique requirements of the military environment, introducing risk assessments to address the particular issues pertaining to military human enhancement.

**Fig. 1:**
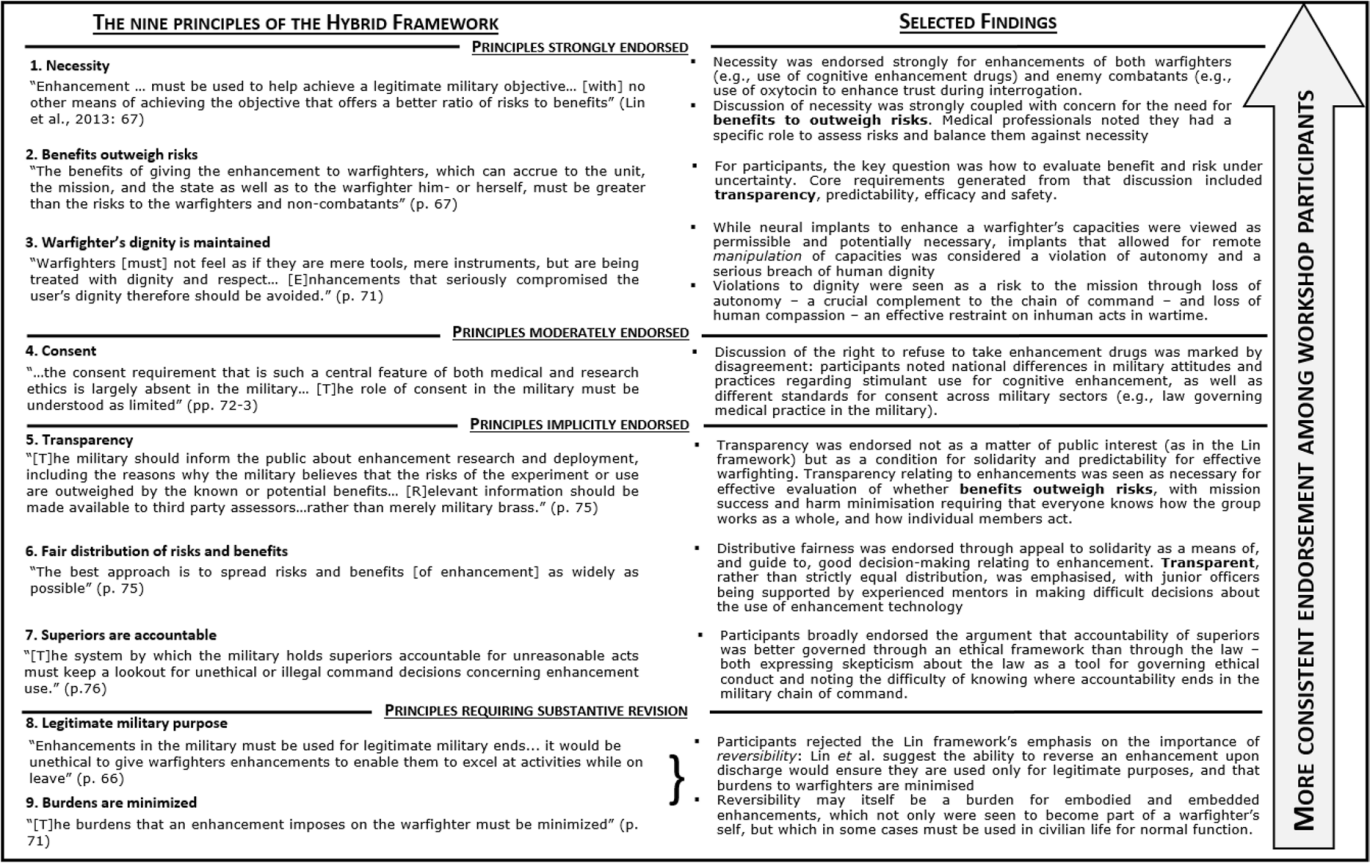
The nine principles of the Lin et al. (2013) Hybrid Framework and the level of endorsement given by workshop participants, including selected focal points of discursive exploration, consensus, and disagreement

The Hybrid Framework consists of nine principles or “rules” (Fig. 1). To our knowledge, despite the key role that the framework has had in influencing thinking in this area, testing development and refinement of the Hybrid Framework has not yet been conducted with the very people who are expected to adhere to and/or implement these (or any other) rules in a specific military context. To address these limitations, we first evaluated the soundness of the Hybrid Framework as part of a set of workshops with military personnel. Based on these findings, we developed a quantitative survey of military officers drawing on vignettes developed in the workshops. Our findings suggest that while there is endorsement of a majority of the Hybrid Framework’s rules, two rules require revision: ‘legitimate military purpose’ and ‘burdens are minimized.’

## Methods Overview

We conducted a two-part empirical investigation with mid-ranking military officers to discuss and explore the ethical dimensions of neuroenhancement in the military. Part one of the investigation involved a series of workshops structured using the set of rules proposed by Lin et al*.* as an analytic guide to explore what military officers thought about the ethical issues that this type of technology might involve. The qualitative results of three structured workgroups informed part two: a digital survey with a larger number of military officers.

### Ethics Statement

Ethics approval was received from King’s College London (ethics approval numbers: MR/14/15–41, MR/15/16–253, MR/16/17–1006, MR/17/18–44, MRA/19/20–14,834) and Oxford CUREC (ethics approval number: R45248/RE001) with gatekeeper approval from the Staff College.

## Study 1: Workshop

### Methods

Three workshops were organized and held at the Joint Services Command and Staff College at the UK’s Defence Academy in Shrivenham. Since 2000, the College has combined the single service provision of the British Armed Forces into a single, purpose-built facility that also draws a significant presence of international officers. Recruitment for the three workshops was aimed at officers taking the Advanced Command and Staff Course at the Lt Colonel (or equivalent) rank. The year-long course is made up of c.280 students drawn from all three services, and while the majority are British, there are also representatives from 40 different countries. Recruitment was done via a general course emailing list supplemented by highlighting by course instructors. There was no pressure or incentive to participate, but as the workshops were held during the volunteers’ ‘free time’, it is likely that there was bias towards those who were specifically interested in the subject to be discussed.

There were no additional selection criteria, and while recruitment numbers were not capped, all three groups (8, 24 and 10) were small enough to permit open discussion and have a free exchange of opinions (the students at the Staff College are very familiar with an environment governed by Chatham House Rules and the non-attribution of individual comments). No recordings of the session were made; however, the research team took turns taking unattributed notes, at least two at a time, which were then compiled. This contributed to accuracy, but the transcript also indicated where the notetakers were less sure of their accuracy. The third author then conducted a broad thematic analysis of the transcript, noting in particular areas of tension between competing values or ethical principles. This analysis was visually summarized for each vignette.

#### Materials and Approach

In the first workshop, participants were given an introductory talk about neuroethics, neuroenhancement and their potential relationship with military developments before being invited to an open discussion about their thoughts, experiences and responses. Following the initial workshop, the research team generated a set of vignettes (see below), based on a combination of the existing neuroethics literature and the experiences documented from the first workshop. The vignettes were used to structure the discussion in an additional two workshops.

The vignettes prompted discussion and debate about scenarios about pharmaceutical enhancement; neural implant enhancement; and neuroprosthetic enhancement, that could arise in a military context (Supplement Information 1). Vignette one was focused on a high-stakes air attack role whether the pilot should take a military-approved controlled stimulant to maintain high alertness over a prolonged period of time. Vignette two involved a soldier issued with a neuroprosthetic limb following a battlefield injury, where the artificial limb is neurologically linked to the user, with the new limb offering increased functionality and strength. The third vignette explored the use of pro social drugs during the interrogation of a failed suicide bomber. Vignette four explored attitudes towards neural implants that enhance sight and hearing, as well as permitting remote monitoring and remote enhancement of cognitive abilities. The themes covered through the vignettes were: right to refuse enhancement in the military context; ethical acceptability of enhancement in the military; consent, reversibility, autonomy, fairness and justice. These vignettes then formed the basis for the quantitative element of the study in part two (see below).

Participants engaged very well in these vignettes, with considered responses both to the case studies themselves and to other participants’ comments. All of the terms employed appeared to be understood by the participants in the workshops and there were no follow up queries or requests for clarification from the survey participants. Participants spoke freely about their own experiences, but personal experience narratives did not feature prominently in the discussion. Officers who had specific expertise, such as medicine or law, spoke from these perspectives, but not exclusively. No participant asked to have any part of the discussion redacted.

### Workshop Results

We have summarized the nine principles of Lin et al*.*’s Hybrid Framework and the degree to which they were accepted/endorsed by the workshop participants in Fig. 1 (Supplement Information 2 for a more detailed description).

The results show that three rules have been strongly endorsed, namely, that: 1) The use of an enhancement must be reasonably necessary to achieve a legitimate military objective. Thereby, an appropriate risk–benefit analysis is required (see next rule) – including the necessity of using enhancements on a warfighter themselves (e.g., should a sleep-deprived long-haul pilot take a stimulant drug) and on an enemy combatant (e.g., should oxytocin be used to enhance trust during interrogation) vs. the risks that are partially unknown (e.g., negative effects on cognition). Here, the medical professionals in the discussion noted that they should be viewed as ‘gatekeepers’ for enhancements. 2) The benefits of enhancements need to outweigh the risks and, transparency, predictability, efficacy as well as safety concerns should guide the assessment of the risk–benefit Eq. 3) Enhancements should not compromise the warfighter’s dignity. For example, implants that allowed for remote *manipulation* of a warfighter’s cognitive or sensory capacities were considered a violation of autonomy and a serious breach of human dignity. Reduction in human dignity was also viewed as a risk to the mission, in two ways: loss of autonomy and loss of human compassion. While warfighters are trained to follow commands and to sacrifice the individual self for the good of the collective and for the mission, autonomous thinking and decision-making was seen as crucial all the way through the chain of command. Workshop participants feared that if combatants were known to be enhanced and/or remote-controlled, a loss of compassion for the enemy could result in treating them less humanely.

Consent as a rule was moderately endorsed. While different legal standards and practices for consent currently exist across military sectors and countries, the right to refuse neuroenhancement was highlighted, also because if the neurotechnology contribute to mission success, it may put pressure on military personnel to consent to its use.

Three rules were indirectly endorsed.^2^ In fact: 1) Transparency about enhancement was seen as a central and important issue and was linked explicitly to several of the other principles such as the effective evaluation of the benefits and risks of enhancement, but also as a condition of solidarity. There should be transparency about who has been enhanced in a mission and how, in order to increase predictability (for mission success, and to minimize harms to warfighters). To this end, comfort with using neuroenhancers would increase if they had been tested on the same individuals before military action, although testing conditions might differ from the field. 2) Officers suggest a fair distribution of enhancements among warfighters, because an unequal distribution can could harm the solidarity in the group. Thereby, fairness can imply equal distribution of risks and benefits, but also a supportive and transparent distribution (i.e., junior officers should not be left to make difficult decisions about the use of enhancement technology, but should be supported by an experienced mentor). 3). Participants neither raised as a concern, nor explicitly discussed, the rule to hold superiors accountable for bullying subordinates into accepting enhancements. However, accountability of supervisors was generally discussed as better being governed through an ethical framework rather than the law (as the law could be used to justify unethical practices and to protect superiors). Thereby, professional and personal integrity should play a major role for knowing where accountability ends in the military chain of command.

Two rules were seen as requiring substantive revision: 1) The rule that enhancement is tied to the concept of legitimate military purpose was seen as problematic, since non-reversibility of enhancements upon leaving the military or while on leave might either constitute a potential ‘burden’ for the warfighter or not be possible. After an enhancement is embedded or embodied, “legitimate military purpose” cannot easily be governed through a distinction between civilian and military life. The warfighter has little choice with regards to virtuously refraining from using an ability provided by a prosthetic hand or visual implant, when on leave. 2) The workshop discussions suggest that the rule to minimize the burden of enhancements (i.e., “discomfort or distress”) should not be uncritically implemented via the expectation of reversibility. As for the prior rule, replacing embedded or embodied enhancement with ‘normal’ prosthetics available (which vary widely across countries) would be unfair. However, officers agreed that enhanced functions (e.g., superior strength) could be problematic in civilian contexts and that expert maintenance of, for example, neuroprosthesis would be challenging. Deservingness and individual character were discussed as potential factors in determining fair distribution of enhancement benefits.

## Study 2: Survey

The survey was designed to further probe the extent of endorsement among military officers for the principles of Lin et al*.*’s Hybrid Framework, and thereby to provide some clear quantitative data to enrich knowledge gained from the workshop phase. The workshop discussions generated 16 items about neuroenhancement pills, neuroimplants, and neuroprostheses for a quantitative web-based survey among military officers from the same location (different cohort).

### Methods

#### Participants and Study Design

We invited military officers from five consecutive cohorts (years 2016–2019) located at the Joint Services Command and Staff College at Shrivenham (UK) to participate in our web-based study.^3^ Participation in our study was completely anonymous and voluntary. A match between the e-mail addresses of the officers and their responses is technically impossible. In total, 1,353 officers were invited, of which 381 (28.2%) started the survey, of these 376 (98.7%) consented to participate, and of which 355 (94.4%) completed the survey.^4^ A small number of civilian civil servants who work with or alongside the military (both in the UK and on operational deployments) were also represented on the courses. We have included them in the full cohort considerations. We excluded individuals who indicated participation in a previous round. Following these exclusions plus list-wise deletion of item nonresponses, the sample of the combined cohorts equals 332 respondents.

#### Instruments

Based on the workshop vignettes, we developed attitude measures to gain a better understanding of the topic in three contexts: pharmaceutical enhancement; neural implant enhancement; neuroprosthetic enhancement. We conducted cognitive think-aloud pretests including probing questions with military officers (*N* = 4) to evaluate the validity, comprehensibility and clarity of our instruments and the instructions prior to the survey. We used the information from these pretests to refine our measures.

##### Attitudes Towards Military Neuroenhancement

We assessed the officers’ attitudes towards enhancement pills with four items, towards neural implants with six items, and towards neuroprostheses also with six items (see item text in Tables 2, 3, 4). Responses were assessed on a scale from 1 “disagree very much” to 7 “agree very much”. To allow a mutual understanding of the topic and the technologies, we provided the officers with brief definitions resembling those in the literature and examples of the technologies and we explained that these technologies might already exist, or that they could appear in the future (Supplement Information 3).

##### Socio-Demographic Information

We assessed the continent where the home military is located as a proximal variable for the cultural, societal, and economic background. Table 1 shows all demographics. Due to the low number of officers outside of Europe, we grouped all non-European officers together. We asked what division the officer was in (e.g., land, sea, air, or civil service). As indicators medical knowledge and deployment experience, we assessed the affiliation to the medical branch and the number of military deployments. In order to maintain anonymity, we were unable assess other demographics such as sex and age.

#### Statistical Analyses

In addition to exploring the level of endorsement regarding the 16 attitudes towards military neuroenhancement descriptively, we used ordered logit regression models [33] to explore how the respondent characteristics are associated with the attitudes. Odds Ratios (*OR*) that exceed 1 indicate that a higher category on an attitude measure is more likely than a lower category, if the value of a variable increases by one unit while the other variables in the model are held constant. *ORs* smaller than 1 indicate that a lower category is more likely, whereas *ORs* equal to 1 imply no effect. Due to space limitations, only statistically significant (*p* < 0.05) effects are discussed in the text. While all models controlled for cohort differences and while we recorded a few inconsistent variations between the five cohorts, we do not discuss these results further. Tables showing the results in greater detail are provided in the Online Supplements (Tables S4, S6, S8).

### Survey Results

#### Enhancement Pills

Our results show that the statement that the military should supply enhancement pills to support mission goals received only moderate support – while 30.7% (very much) agree, also 17.8% (very much) disagree. This is also indicated by a relatively large standard deviation (Table 2). Especially those from the sea component disagreed more in comparison to land (Model 1, Supplementary Information 4 and Table 5 that summarizes all findings from the regression analysis). There was more approval regarding whether military personnel should be allowed to refuse an order to take enhancement pills. Here, 74.1% (very much) agreed. Agreement was lower in the land component in comparison to air. The higher the number of deployments, the lower the agreement. Especially strong approval and high consensus can be seen with regard to whether military personnel should be allowed to freely decide about participation in military research on enhancement pills. Here, 91.0% (very much) agreed to this statement. All seven civil servants in the sample agreed very much to this statement (no estimation of the effect was possible due to the homogeneity of responses, Model 3). The majority of officers (77.2%) (very much) agreed that the military should conduct research to test the safety and efficacy of enhancement pills. Officers from the sea component showed considerably less agreement compared to officers from the land and the air component (Model 4).

An exploration of the correlations between attitudes towards enhancement pills reveals, for example, a substantial positive association between the approval that the military should supply such pills for mission goals and that their safety and efficacy should be tested (*r* = 0.39, *p* < 0.001, Supplement Information 5). Officers who strongly endorsed the supply by the military, however, were less supportive of allowing personnel to refuse orders to take such pills (*r* = -0.24, *p* < 0.001). Those officers who demonstrated a stronger approval for permitting individual refusal, also felt that the decision to participate in research on such pills should be voluntary (*r* = 0.22, *p* < 0.001), but less that the military should test drug safety and efficacy (*r* = -0.14, *p* < 0.05).

#### Neural Implants

Similar to the high agreement about whether military personnel should freely decide about participation in military research on enhancement pills, a majority of 88.8% (very much) agreed to this with regard to neural implants (Table 3). But agreement was lower with more deployments (Model 1, Supplement Information 6). All officers from the civil service component agreed very much to this statement. Almost all (96.1%) officers (very much) agreed that military personnel should be informed prior to surgery whether they can keep a military-supplied neural implant after leaving the military – especially those from European countries compared to those from outside of Europe (Model 2). As indicated by the large standard deviations, respondents’ views relatively strongly diverged on whether the military should base decisions about the removal of neural implants on whether the implant treats a condition or enhances functions. On average the support for this principle can be seen as moderately strong – 46.7% (very much) agreed to this, while 14.7% (very much) disagreed. Opinions on whether enhancing neural implants provided to the military service is the property of the military are also highly diverging as indicated by the standard deviations; 23.9% (very much) agreed to this, while 26.5% (very much) disagreed. Similarly, mixed opinions exist on whether it is dangerous if a soldier would keep an enhancing neural implant after leaving the military: 17.4% (very much) agreed to the statement, while 22.0% (very much) disagreed. Officers from a European country disagreed more than those from a non-European country (Model 5). A majority of 71.1% (very much) agreed that veterans who lost eyesight or hearing as a result of a mission should be prioritized for neural implants that allow normal functioning.

Support for free decision-making about participation in military research on neural implants was positively associated with both endorsement of the view that personnel should be informed whether they can keep enhancing implants after leaving the military (*r* = 0.27, *p* < 0.001, Supplement Information 7), and the view that veterans who were physically impaired during missions should be prioritized for implants (*r* = 0.14, *p* < 0.01). The latter two attitudes were also positively correlated (*r* = 0.20, *p* < 0.001). Moreover, positive associations were found between further three attitudes: support for basing decisions about implant removal on the basis of the treatment-enhancement distinction correlated with both approval that enhancing implants should remain military property (*r* = 0.47, *p* < 0.001), as well as the approval that granting such implants to soldiers after their leave is dangerous for society (*r* = 0.42, *p* < 0.001). The latter two attitudes were also correlated (*r* = 0.51, *p* < 0.001).

#### Neuroprostheses

Generally, the descriptive findings regarding all attitudes towards neuroprostheses are comparable to those for neural implants. As for the other enhancement technologies, there is high agreement and consensus that military personnel should freely decide about participation in military research on neuroprostheses – 91.0% (very much) agreed to this (Table 4), including all from the Civil Service component (Model 1, Supplement Information 8). The higher the number of deployments, agreement decreased. Support on whether military personnel should be informed prior to surgery whether they can keep a military-supplied neuroprostheses implant after leaving is similar to the same issue regarding neural implants – 96.4% (very much) agreed. Similar to the views on neural implants, support is moderately strong and relatively strongly diverging on whether the military should base decisions about the removal of neuroprostheses on whether the prosthesis treats a condition or enhances functions. About half (46.1%) of the officers (very much) agreed to the statement, whereas more than every tenth (13.2%, very much) disagrees. Opinions on whether enhancing neural implants provided to the military service is the property of the military are also highly diverging: 25.6% (very much) agreed to this, while 27.8% (very much) disagreed. Opinions on whether it is dangerous if a soldier would keep an enhancing neuroprosthesis after leaving the military are mixed: 16.2% (very much) agreed, while 23.8% (very much) disagreed. The plurality of 72.6% (very much) agreed that veterans who lost eyesight or hearing as a result of a mission should be prioritized for neuroprostheses that allow normal functioning. Officers from the civil service component agree more to this than those from the air component (Model 6).

The pattern for the correlations between attitudes towards neuroprostheses are identical to those reported for neural implants (Supplement Information 9).

## Discussion

Our research sheds some light on the broader extent of endorsement of the Lin et al. Hybrid Framework among military personnel, and how its principles might be realized in practice. For example, we found moderate to high agreement regarding the Hybrid Framework rules about the supply of, and the need to conduct research into enhancement pills in the military. Survey findings endorsed the importance of decision-making autonomy of military personnel both to refuse orders to take such pills, and whether or not they participated in research into them. Similarly, decision-making autonomy about whether to participate in military research about neural implants and neuroprostheses was strongly endorsed. Also endorsed was the need for information about whether the technologies can be kept after leaving the military. Additionally, there were high levels of agreement that veterans who are impaired during a military mission should be prioritized when the technology reaches the civilian market. There was more divergence in opinion regarding the removal of the enhancing vs. treatment-focused devices, property rights and perceptions of danger for society after military service.

### High Importance of Decision-Making Autonomy and the Consideration of Benefits and Risks

Across all three neuroenhancement technologies, there were high levels of support for the individual warfighter’s decision-making autonomy – effectively recognition of the importance of the principle of consent in the context of neuroenhancement technologies. This is in spite of an acknowledged sense that, in military contexts, “the hallmark principles that drive bioethical decision making in ordinary clinical settings are largely absent. Military personnel do not enjoy a right to life, personal autonomy, or a right of self-determination to any degree approaching that of ordinary patients” [34]. Interestingly, for some respondents, decision-making autonomy might be constrained as soon as they endorsed a supply of neuroenhancing pills to support mission goals, perhaps reflecting a view that their military utility was so high, that individual choice was not an appropriate barrier to military necessity. However, workshop participants’ endorsement of the need to balance risks and benefits, alongside correlation results between the attitudes, suggest that advocacy for the supply of neuroenhancing pills is accompanied by calls for more research on them. Such sentiments may be driven by a recognition that trade-offs may exist between enhancing performance in one operationally-relevant domain to the cost of performance decrements in another [19, 35].

### Transparency and its Impact on the Moral Priority of Reversibility

In the workshops, transparency was prioritized and confidentiality was not seen as a virtue. This reflected the view that there should be no secrets among warfighters working together on a mission as to who was enhanced and how. The absence of secrecy was strongly related to the requirement of predictability: for mission success, and to minimize harms to warfighters, everyone needs to know how the group works as a whole, and how individual members will act. Similarly, survey respondents consistently agreed that, before surgery, warfighters should be told whether they can retain neuroenhancements post-discharge. As well as suggesting a respect for the ‘clinical’ bioethical principles of *informed* consent, this is consistent with various aspects of the Hybrid Framework: namely, the importance of *transparency* (although, between the military and an individual warfighter, rather than between the military and the public), as well as *dignity*: ensuring full information about a neuroenhancement protects against a warfighter experiencing him or herself as a tool or weapon to be augmented and diminished at the whim of the military. This latter concern also came out strongly in the workshops, especially with reference to technology that could reduce autonomy and could lead to military personnel being perceived as either ‘rats’ or ‘robots’. The high endorsement for ensuring transparency suggests that, *pace* Lin et al*.*, efforts to minimize the burdens associated with neuroenhancement should not be interpreted through the simple proxy of reversibility. For example, from the workshops it was clear that those enhancements which were connected to, and enabled via the brain, such as neural prosthetics, were seen to become part of a person’s ‘self’, and that it would be unfair to replace these with ‘normal’ prosthetics available in the country’s public health service. This accords with research done at Case Western Reserve University which noted the unexpected but intense grief exhibited by test subjects when they were required to return advanced prototype prosthetic hands after testing, with some describing this as worse than losing their hand the first time [36]. The comparatively low levels of agreement among survey respondents that military-issued neuroenhancements remain the property of the military likewise suggests that respondents did not consider the technologies a burden for which reversibility is a moral priority.

### Principle of Necessity in the Context of Post-Military Neuroenhancement

Although responses were divergent, the moderate endorsement of using the therapy-enhancement distinction to base decisions about neuroenhancement removal suggests limited acceptance of the framework’s principle of necessity in the context of civilian life after military service. Moreover, agreement with these survey items for both neuroimplants and neuroprostheses positively correlated with a perception that it is dangerous to society for former soldiers to retain neuroenhancements after service, reflecting an implicit endorsement of the Hybrid Framework’s principle that benefits must outweigh risk. The results point towards some caution of the respondents since technologies with enhancement properties might be more dangerous and therefore, they were likely expressing their support for the technology remaining in military possession.

### Contextual Factors in Attitudes

We did not find large variation in attitudes by socio-demographic make-up of the participants. Airforce officers were more likely to support refusal of orders to take enhancement pills than officers from the land; and all civil service officers endorsed autonomous decisions about research participation in all three forms of neuroenhancement. With more deployments, officers granted less decision-making autonomy about whether military personnel should be allowed to freely decide about participation in military research on implants and neural prostheses as well as to refuse orders to take enhancement pills. The reasons for these differences remain unclear, but it may be linked to increased tendencies towards hierarchical thinking: a greater number of deployments may influence personnel’s acculturation into military culture, or the extent to which their self-identity is bound together with their military role. Alternatively, more deployments are likely to correlate with age, with more advanced aged perhaps linked to a greater prevalence of thinking in hierarchies.

### Limitations of our Approach

This study gathered insights into attitudes to neuroenhancement from a currently under-investigated, but highly relevant target group. Due to the lack of data in the literature, we developed a quantitative survey using an initial qualitative approach to ensure relevance and validity of survey vignettes and questions. Over repeated survey waves, the response rate remained relatively low, which might be explained by the tight schedule of the officers during their time at the Academy. Due to significant access challenges to this population, our initial decision was to focus this first study of its kind on the well-informed, well-educated mid-ranking officers who are likely to have been exposed to a wider overview of defence matters due to their career profile and prospects. As such, the sample is not representative of the military as a whole, but rather offers a starting point by examining the perspectives of those likely to be in command positions and therefore positions of responsibility as neuroenhancements become more ubiquitous. Future studies should try to access a more representative military sample, including enlisted personnel, and increase the number of participants, e.g., by providing (non-)monetary incentives [37, 38]. We also had a low proportion of officers outside Europe, in the Civil Service component, and in the medical branch, which limits the generalizability of our findings. We tried to reduce social desirability bias by guaranteeing anonymity [39]; however, this prevented assessment of the impacts of potentially identifying features in the target population, such as age and gender. Future studies may, however, try to assess whether further demographics result in homogeneous or heterogenous evaluations of neuroenhancements across demographic groups. Also, prior personal or vicarious exposure to neuroenhancements would be a candidate factor for further exploration of attitudes towards neuroenhancements. Moreover, combat experience could be an interesting indicator for officers perceived need of neuroenhancement means before, during, or after combats. Another avenue for further research to better understand the variation in military officers’ attitudes to neuroenhancement could be to examine how the “ethical framework” [40] of the officers, i.e., their learned preferences about “how to behave, judge or solve moral problems” [41] co-varies with their attitudes to neuroenhancement. For example, their preferences for precepts implied in moral theories (PPIMT), namely “virtue ethics (e.g., “strive to be an honest person”), deontology (e.g., “obey rules, such as ‘never lie’”), and consequentialism (e.g., “maximize happiness and save lives with any means necessary”)” may guide their judgement either consciously or unconsciously [41].

## Conclusion

Having gathered the perspectives of staff officers on the abstract principles of Lin et al*.*’s Hybrid Framework through a series of workshops, we sought to enrich our understanding of these attitudes by gathering data from a larger group of military officers through a survey. By collecting quantitative measures of endorsement for a number of concrete decision-making guidelines, we were able to generate an overview of how mid-ranking military officers might, in practice resolve tensions between competing values or higher-level principles.

Perhaps unsurprisingly given the extent of disagreement within the military ethics literature regarding consent, perspectives on the matter of decision-making autonomy for individual warfighters were found to be diverse and context-specific in both the workshop and survey elements of the study. Disagreement about right-to-refuse military-supplied enhancement pills was found in open discussion, and was reflected in the comparatively large standard deviation in responses to the respective survey item. The survey found stronger consensus in favour of right-to-refuse participation in experimental military research for neural implants and neuroprosthetics as well as for enhancement pills. This difference likely reflects the need for warfighters to submit to orders from commanding officers while on active deployment. Moreno notes, for example, that the US Uniform Code of Military justice requires soldiers:

“to accept medical interventions that make them fit for duty. Experimental treatments are a harder case, but the US government has shown a tendency to defer to commanders in a combat situation if they think some treatment is likely to do more harm than good, even if unproven.” [23].

It remains to be seen how military neurotechnologies, and the discourse concerning them, will develop, but the potential for enhancements to be normalized as a standard aspect of warfare demands further attention be paid to navigating the appropriate role of consent in military contexts. The requirement to inform warfighters in advance about the retention of neural implants or neuroprostheses was unanimously endorsed, with no respondent expressing even a slight disagreement. Given the workshop participants’ insistence that it would be unfair to demand that warfighters give up enhancements after service, these very strongly endorsed survey items suggest that post-service retention of enhancements may be an appropriate means to ensure the fair distribution of risk and benefit. However, it remains unclear what drives this sentiment. Lin et al. note that there is no significant ethical discourse surrounding traditional, external enhancements such as body armour [22]. Since there is likely no parallel objection to warfighters returning military-issued body-armour after service, the precise grounds of this difference require further articulation. The difference may be driven by the comparative ease of reversibility of body armour and a neuroenhancements, distinct rates of risk and benefit for those being issued the enhancement, or perhaps a sense in which an embodied or embedded enhancement comes to be seen as part of the self—an issue that was raised in all three of the workshops. Allowing demobilised warfighters to keep their enhancements opens new questions about the level of ability ‘owed’ to veterans as they return to civilian life, and about the scope of the military’s responsibility to take care of veterans as embedded enhancements might require maintenance [27].

The insistence from workshop members that it would be unfair to demand that enhancements are given up after service suggest that the Hybrid Framework’s principle of legitimate military purpose could be revised to include the corollary of legitimate dual use, permitting actions both within and without military service, perhaps under certain conditions. Across all survey items, the suggestions that military-supplied neuroenhancements remained military property, or that enhanced warfighters would pose a danger to society after service, received the lowest levels of support of any items.^5^ However, these items also received among the lowest levels of consensus. The diversity of sentiment surrounding the status and risk of neuroenhancements in post-deployment civilian contexts, underlines our suggestion that the introduction of these technologies in a military context demands a recontextualisation of the relationship between military and civilian ethics.

In sum, our study suggests that more clarity, more information, more evidence and more guidance will support this re-contextualisation and enable officers to uphold a duty of ethical conduct that they consider more important than ever in the current climate.

**Table 1:** Sample descriptives (*N* = 332)

**Categorical measures**	*Absolute*	%
*Location of home military*		
• Africa	3	0.9
• Asia	16	4.8
• Australasia	5	1.5
• Europe	293	88.3
• North America	13	3.9
• South America	2	0.6
*Component*		
• Land	143	43.1
• Sea	99	29.8
• Air	83	25.0
• Civil Service	7	2.1
*Medical branch*		
• Yes	15	4.5
• No	317	95.5
*Cohort*		
• 1	66	19.9
• 2	81	24.4
• 3	75	22.6
• 4	50	15.1
• 5	60	18.1
**Continuous measure**	*Mean (SD)*	*Min/Max*
*Number of deployments*	6.79 (6.00)	0/50

*SD* Standard deviation, *Min* Minimum, *Max* Maximum

**Table 2:**
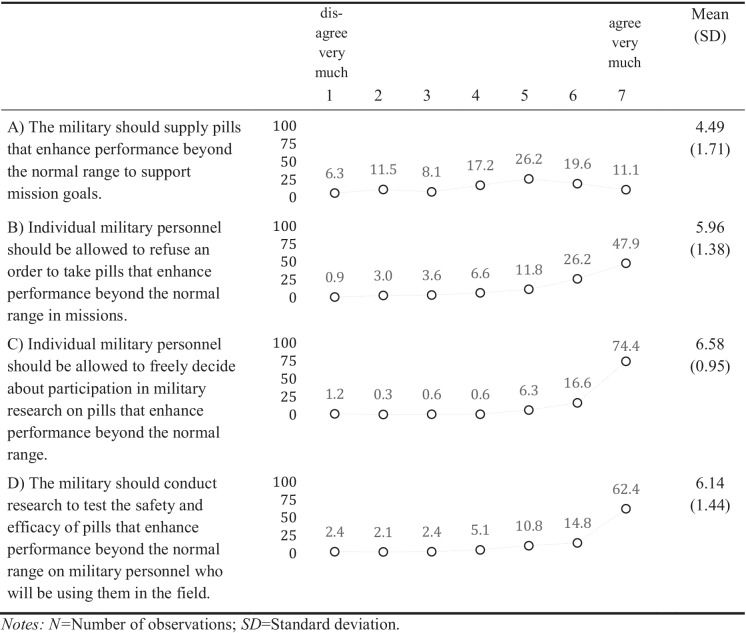
Descriptive statistics of the attitudes about enhancement pills (% of responses, *N* = 332)

**Table 3:**
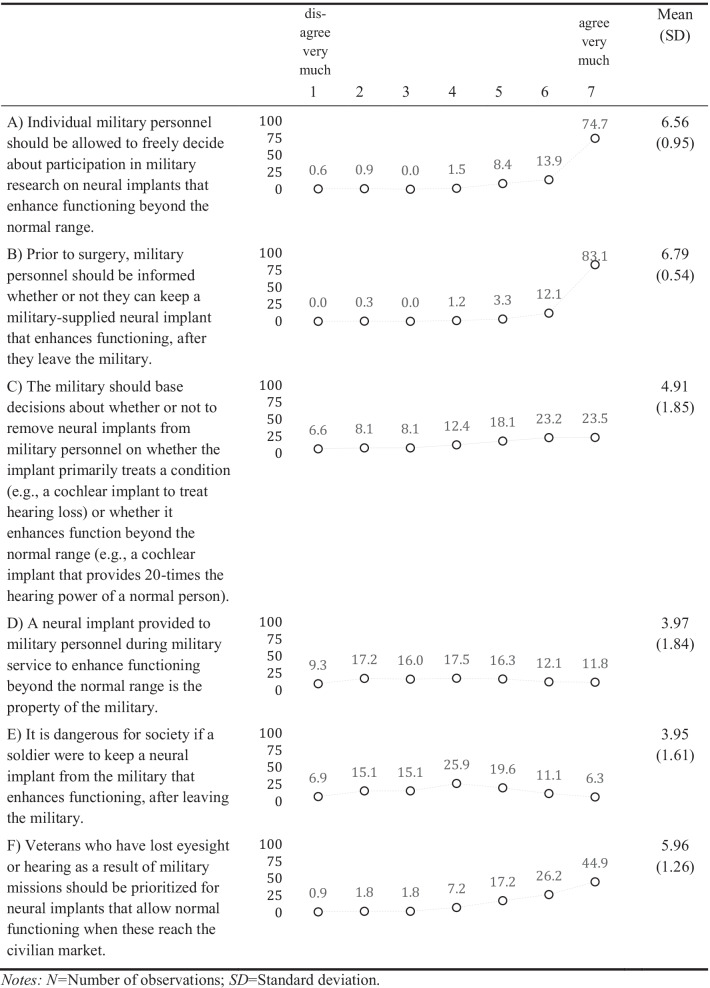
Descriptive statistics of the attitudes about neural implants (% of responses, *N* = 332)

**Table 4:**
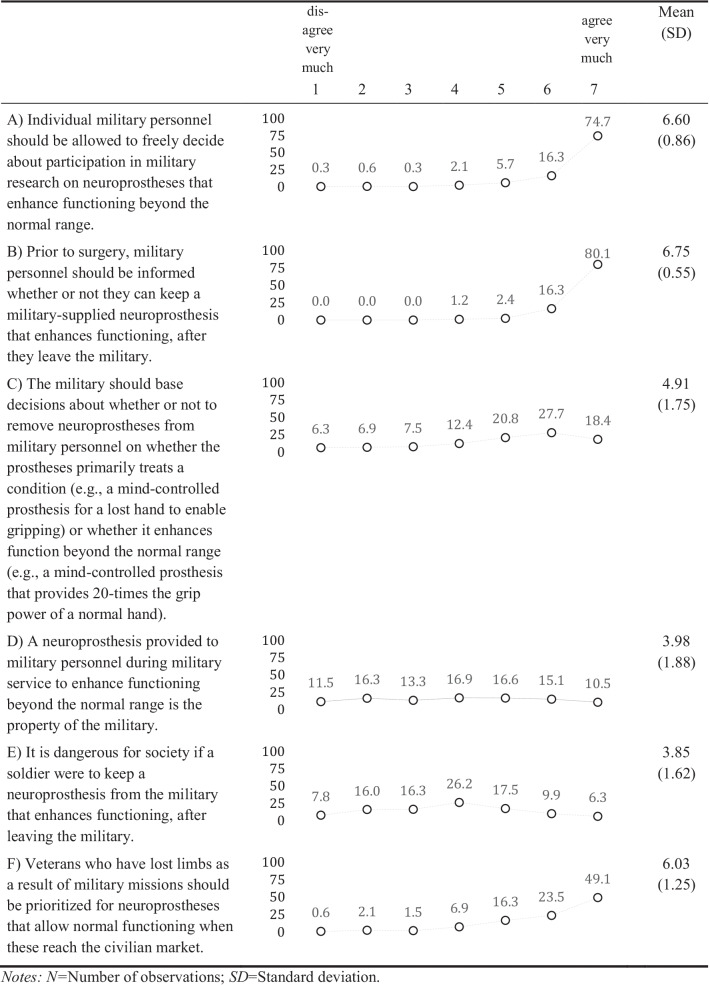
Descriptive statistics of the attitudes about neuroprostheses (% of responses, *N* = 332)

**Table 5:**
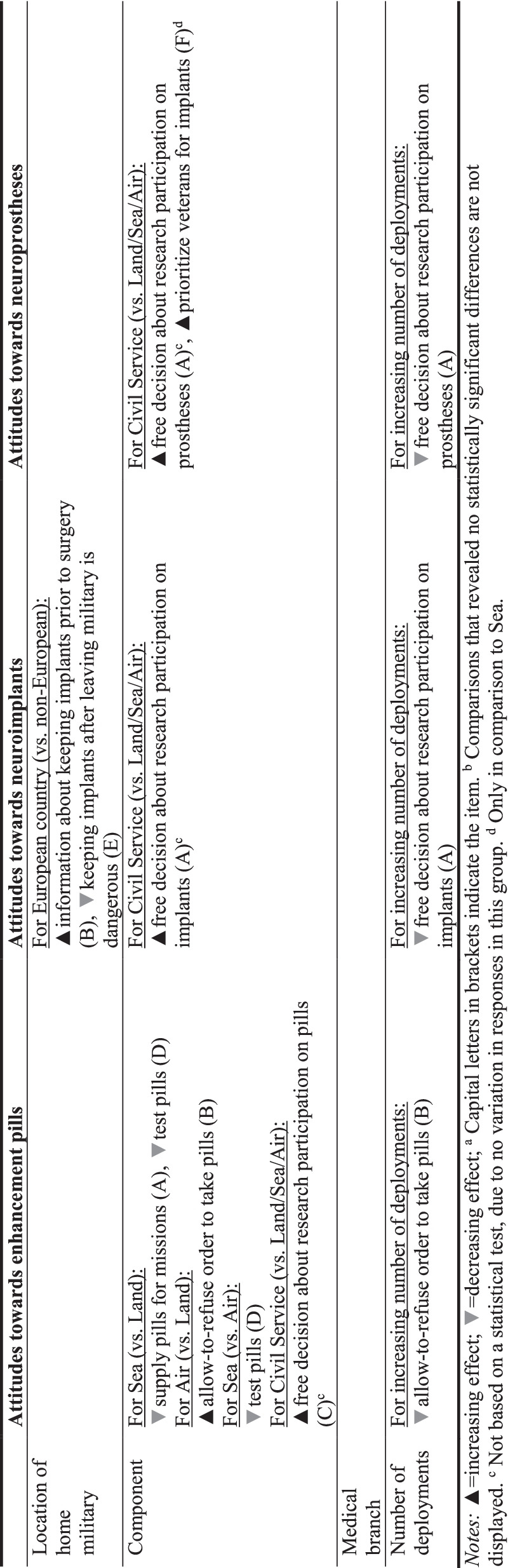
Summary of the findings^a,b^

## Supplementary Information

Below is the link to the electronic supplementary material.

Supplementary file1 (DOCX 81.6 KB)

## Data Availability

The data cannot be deposited. The research material has been extensively described in the manuscript and the Supplementary Information.

## Footnotes

1 This appears to have been substantially influenced by a recent academic publication (June, 2020) titled, “Enhancing Soldiers: A European Ethical Approach,” edited by Gérard de Boisboissel and Magdalena Revue, under the auspices of the Saint Cyr Military Academy Research Centre and the International Society for Military Ethics in Europe [31].

2 An ‘indirect endorsement’ is one that does not cohere with Lin et al.*’s* definition or examples, but nevertheless agrees with the spirit of the rule.

3 Officers from the first cohort received an invitation email detailing the information, followed by two email reminders. In the second and third cohort, a prenotification letter detailed the information about the participation, followed by an invitation email and one email reminder, while the fourth and fifth cohort only received an invitation email and two email reminders. There were two cohorts in 2017.

4 Numbers by cohort: N_INVITED-1_ = 272; N_INVITED-2_ = 283; N_INVITED-3_ = 271; N_INVITED-4_ = 257; N_INVITED-5_ = 270; N_STARTED-1_ = 83; N_STARTED-2_ = 95; N_STARTED-3_ = 86; N_STARTED-4_ = 55; N_STARTED-5_ = 62; N_CONSENTED-1_ = 79; N_CONSENTED-2_ = 94; N_CONSENTED-3_ = 86; N_CONSENTED-4_ = 55; N_CONSENTED-5_ = 62; N_COMPLETED-1_ = 72; N_COMPLETED-2_ = 87; N_COMPLETED-3_ = 79; N_COMPLETED-4_ = 55; N_COMPLETED-5_ = 62.

5 The perceived risk of enhanced warfighters posing a danger to society after service may, in part, be a function of perceptions of broader societal and political stability. Notably, the January 2021 storming of the US Capitol had a significant veteran presence, with as many as 20% of those charged following the attack having a military history [42].
