# Supplementary material for: Neuroenhancements in the Military: A Mixed-Method Pilot Study on Attitudes of Staff Officers to Ethics and Rules
Source: Neuroethics. 2022 Feb 28;15(1):11. doi: 10.1007/s12152-022-09490-2 (PMC8885476; doi:10.1007/s12152-022-09490-2)
Supplement: Supplementary file 1 — Supplementary file1 (DOCX 81.6 KB) [file 12152_2022_9490_MOESM1_ESM.docx]

Supplementary Information

“**Neuroenhancements in the Military: A Mixed‑Method Pilot Study on Attitudes of Staff Officers to Ethics and Rules**” by Sebastian Sattler, Edward Jacobs, Ilina Singh, David Whetham, Imre Bárd, Jonathan Moreno, Gian Galeazzi, Agnes Allansdottir. Published in *Neuroethics*: https://doi.org/10.1007/s12152-022-09490-2

**Supplement Information 1.** Case vignettes of study 1.

| **Technology** | **Vignette** | **Guiding questions** |
| --- | --- | --- |
| Enhance-  ment pills (performance) | Oliver has recently completed his flight combat training and is about to be deployed. He is informed that due to increased tensions in the Middle East, he must engage in a high-stakes ground attack mission in northwestern Syria. He will need to maintain high alertness and precision over a 24-hour period. Oliver’s medical pack contains amphetamines, which he knows some pilots use on missions like this. | - Should Oliver take the tablets? - What if Oliver’s commanding officer orders him to take the tablets? - What if Oliver’s fellow pilots ask him to take the tablets? - Is there a non-military context in which employers would be justified in asking employees to take stimulant drugs? |
|  |  |  |
| Enhance-  ment pills (morals) | A class of morally-enhancing drugs is created that increases empathy and pro-social behavior. The UK government has decided to use these drugs on operations to increase compliance among known terrorists who have been captured and are being interrogated in order to gather intelligence and prevent further major crimes against the civilian population. Chris is a junior officer who has been put in charge of the drug-assisted interrogation of a failed suicide bomber in order to find out where the suicide vests are being assembled, and by whom. It is known that the vests are being made by the same bomb-maker who has been responsible for over 200 civilian deaths this week alone. An older, more experienced peer tells Chris that prisoners often need to be force-fed the drugs, as they otherwise refuse to take them. He also reminds Chris that lives are at stake and other, more robust, interrogation methods remain an option. | - What should Chris do? - Torture is illegal, but if the administration of morally enhanced drugs have been approved by UK government lawyers, does that make it ok? - Is there actually a difference between using these drugs and using more forceful methods for interrogation? - Is there an ethical difference (if we understand ethics to be a general set of principles that guide the behavior of social groups)? - Should morally enhancing drugs be used outside the military? |
|  |  |  |
| Neural implants | It’s 2040 and Western military scientists have developed neural implants that allow soldiers to be more efficient and effective fighting machines. One set of neural implants significantly improves and expands a soldier’s sight and hearing capacities, such that surveillance of people, communities and objects is possible in low-light conditions, at night, and over long distances. Another set of neural implants allows remote monitoring of a soldier’s cognitive patterns. When it is deemed appropriate, the soldier’s cognitions can be remotely influenced allowing him to process information faster, analyze situations at a glance etc. While the soldier is aware of the implant, and that his cognitive processes are being monitored, he cannot know which of his thoughts and decisions are the result of remote input. The military predicts that by 2050 a majority of military personnel on the ground will have one or the other set of neural implants (the neuro-pathways cannot work with both). However, each soldier must willingly consent to the implant, and each soldier can choose which kind of implant to have. | - Is there a difference between the two types of neural implant? - Are their justifiable reasons for a soldier to refuse one or the other kind of implant? - Does either implant make a soldier less human? - Is there a non-military context in which such implants would be useful or necessary? |
|  |  |  |
| Neuro-prostheses | While deployed in Iraq, Christopher lost his arm, to the elbow, to a roadside bomb. After recovering from the injury, Christopher expressed a strong desire to return to combat. The British Armed Forces provided him with a state-of-the-art neuroprosthetic arm, which allows him to control his movements with his mind. The arm has several advantages over a human arm; its built-in sensors allow remote tracking of Christopher’s physiological signs and movements, and remote deployment of weapons. The prosthetic hand has six multi-directional fingers that have the strength of many human hands and double as tools. Christopher completed several tours of duty successfully with his neuroprosthesis, and is about to be discharged from the army. He learns that upon discharge, he will be given a standard NHS prosthesis with limited functionality and a less aesthetic appearance. | - Is it fair that Christopher will get an ordinary prosthetic arm after his discharge from the military? - Does Christopher have any rights over a neuroprosthetic limb that he has worn for several years? Does it matter that this is a neuro- prosthesis – that is, linked to his brain? - What is the military’s responsibility, if any, to ensure that the technology they have developed for the military-grade neuroprostheses is accessible to the general public? |

**Supplement Information 2.** Extensive description of the workshop results (Part 1).

| **Level of endorse-ment** | **Principle** | **Results** |
| --- | --- | --- |
| Strong | Necessity | The rule that the use of an enhancement must be reasonably necessary to achieve a legitimate military objective was endorsed by the workshop participants, but the concept was strongly coupled to appropriate a risk-benefit analysis (see also next section), as underlined by Lin et al. (2013), and also by Gross (2006). This was true both for the use of enhancement on a warfighter himself (e.g., use of cognitive enhancement drugs), and use of an enhancement on an enemy combatant (e.g., use of oxytocin to enhance trust during interrogation). Rather than engage in an analysis of necessity as an independent factor, the discussion among participants continuously moved between considerations of necessity (should a sleep-deprived long-haul pilot take a stimulant drug) and considerations of risk and benefit (to what extent was this pilot’s individual response to the drug known; how much was known about the condition of the other pilots flying with him; could the drug have negative effects on cognition etc.). The participants in the discussion who were also medical professionals noted that they should be viewed as ‘gatekeepers’ to drugs used for enhancement, and that they had a specific role to assess risks and to balance these against necessity. |
| Strong | Benefits outweigh risks | The rule that benefits of enhancements need to outweigh the risks was absolutely endorsed by the workshop participants, with a good deal of discussion oriented around the balance of benefit and risk surrounding enhancements. For the participants, the key question was how to make a good assessment of the risk-benefit equation. The discussion illuminated a set of core requirements that guided the evaluation of benefit and risk in conditions of uncertainty: transparency, predictability, efficacy and safety. |
| Strong | Dignity is maintained | When debating the use of technology that might allow remote monitoring or manipulation of a warfighter’s cognition and/or emotions, the workshop discussions most directly endorsed the rule that enhancements should not compromise the warfighter’s dignity. While the use of neural implants to enhance sensory capacities, or to overcome physical limitations was viewed as potentially necessary in the context of military activities, implants that allowed for remote manipulation of a warfighter’s cognitive or sensory capacities was considered a violation of autonomy and a serious breach of human dignity. Workshop participants likened warfighters subjected to the latter technology as “rats” or “robots.” Reduction in human dignity was also viewed as a risk to the mission, in two ways: loss of autonomy and loss of human compassion. Participants were clear that while warfighters are trained to follow commands and to sacrifice the individual self for the good of the collective and for the mission, autonomous thinking and decision-making is nevertheless crucial all the way down the chain of command. Loss of compassion was linked to a change of perception when humans “act more like robots.” Compassion for the enemy as a fellow human being was seen as an effective restraint on inhuman acts in war time. Workshop participants feared that if combatants were known to be enhanced and/or remote-controlled, there was a risk that they would be treated less humanely. |
| Moderate | consent | Consent as a rule was strongly endorsed. However, the vignettes presented to the workshops did not address scientific experiments involving human military subjects. Rather, consent was invoked in a discussion about stimulant drug use to enhance alertness, and in a discussion about the right to refuse to interrogate an enemy warfighter using oxytocin on moral grounds. In the discussion around stimulant use for enhancement, participants disagreed about whether military personnel should have the right to refuse to take stimulant drugs. Participants noted that there are different legal standards for consent across military sectors: for example, participants who had military medical roles felt that they were required to follow laws that govern medical practice in the military. Moreover, there were national differences in military attitudes and practices around the use of stimulants as cognitive enhancers, with some countries having a laxer attitude than others (outside considerations of lawfulness). Participants debated whether this fact put pressure on military personnel to consent to stimulant drug use, particularly if this use were shown to contribute to mission success. |

**Supplement Information 2.** Extensive description of the workshop results (Part 2).

| **Level of endorse-ment** | **Principle** | **Results** |
| --- | --- | --- |
| Indirect | Transp-arancy | Transparency about enhancement was raised as a central and important issue in the discussions and was linked explicitly to several of the other principles (see above). It was typically discussed with regard to effective evaluation of benefits and risks of enhancement, and as a condition of solidarity rather than as concern about public transparency (which is the focus of the Hybrid Framework definition). Transparency was discussed in relation to group dynamics and the role of the individual within a group comprising a military team. In the group context, transparency was prioritized and confidentiality was not seen as a virtue. This reflected the view that there should be no secrets among warfighters working together on a mission as to who was enhanced and how. Lack of secrecy was strongly related to the requirement of predictability: for mission success, and to minimize harms to warfighters, everyone needs to know how the group works as a whole, and how individual members will act. Participants noted that they would feel more comfortable with the use of neuroenhancers if they had been tested on the same individuals before military action, despite the fact that such testing might not adequately capture conditions in the field. |
| Indirect | Fair distribution of risks and benefits | The importance of distributive fairness was implicitly endorsed in the frequent calls on solidarity as a means of, and a guide to good decision-making in relation to enhancement. However, fair distribution of risks and benefits in the context of solidarity does not necessarily mean equal distribution: the workshop discussions focused rather on supportive and transparent distribution. For example, participants felt that junior officers should not be left to make difficult decisions about the use of enhancement technology; fair distribution in this context would provide the junior officer with an experienced mentor to support him or her. |
| Indirect | Superiors are accountable | Participants did not specifically discuss accountability of supervisors for bullying others into accepting some form of enhancement, and it was not raised as a concern. This may reflect the relative seniority of the officers; it would be interesting to compare these attitudes to those of younger, less experienced military personnel. In general, participants endorsed the argument implicit in the Hybrid Framework, that accountability of superiors was better governed through an ethical framework than through the law. Participants expressed a lot of skepticism about the law as a tool for governing ethical conduct and accountability in the context of military uses of enhancement. Some practical issues were discussed: in a military context, the law could be used to justify unethical practices and to protect superiors. The law requires individual culprits, and participants discussed the difficulty of knowing where accountability ends in the military chain of command. In view of these difficulties, the officers highlighted professional and personal integrity as the key to accountability of superiors. |

**Supplement Information 2.** Extensive description of the workshop results (Part 3).

| **Level of endorse-ment** | **Principle** | **Results** |
| --- | --- | --- |
| Sub-stantive  revision needed | Legitimate military purpose | The workshop discussions highlighted the problem that, once enhancement is embedded or embodied, “legitimate military purpose” cannot easily be governed through a distinction between civilian and military life, because the same human body co-exists in both contexts. This is true for non-technological enhancements as well: a warfighter whose body has been trained for extreme physical endurance, for example, retains that ability when on leave. For specific martial arts skills, such as hand-to-hand combat, the situation is more pronounced. Privately, the warfighter must choose to adhere to the rule of “legitimate military purpose,” a choice that entails what Lin et al. (2013) call “character and virtues” rather than abusing this skill. The exceptional issue with some embedded or embodied enhancement technologies is that the warfighter may not have this choice to act virtuously. To function normally while on leave, the warfighter has no choice but to use her prosthetic hand or visual implant and would therefore normally excel at some civilian activities without effort, even though excellence in civilian life was not the primary motivation for enhancement. |
| Sub-stantive  revision needed | Burdens are minimized | The workshop discussions suggest that the rule to minimize the burden of enhancements should not be uncritically implemented via the expectation of reversibility. In fact, reversibility may itself be a burden of some neuroenhancements in the military. Different kinds of neuroenhancements were discussed in relation to reversibility. For example, it was clear that those enhancements which were connected to, and enabled via the brain, such as neural prosthetics, were seen to become part of a person’s ‘self.’ Workshop participants thought it would be unfair to replace these with ‘normal’ prosthetics available in the country’s public health service (the kinds of prosthetics available would vary widely). However, it was not necessarily thought to be appropriate to continue to provide a war fighter with enhanced functions upon leaving military; furthermore, these enhanced functions, such as superior strength, could be problematic in civilian contexts. Pragmatic concerns were also raised: expert maintenance of such a prosthesis would be challenging to locate in civilian life, and could not be expected over the long term, unless the military continued to pay for prosthetic upgrades and maintenance. Deservingness and individual character were discussed as potential factors in determining fair distribution of enhancement benefits in particular; an example that generated discussion was soldiers who lost limbs due to carelessness or not following orders, rather than in combat situations. Some wondered if these soldiers should receive lower priority for military grade prosthetics. |

**Supplement Information 3.** Definitions of enhancement technologies provided the military officers.

| **Technology** | **Short definition** |
| --- | --- |
|  |  |
| Enhancement pills | Pharmaceutical agents that can enhance different functions of the brain beyond the normal range such as concentration or memory. |
|  |  |
| Neural implants | Technologies that are connected to the brain and can serve as replacements for normal functions, or they can enhance functions beyond the normal range. Examples include retinal implants and cochlear implants, which can replace dysfunctional sight and hearing, or can enhance sight and hearing beyond what is normal in human beings. |
|  |  |
| Neuroprostheses | Technologies that are connected to the brain and can serve as functional replacements for e.g., lost arms, legs or hands. Neuroprostheses can also serve to enhance functions beyond the normal range, thereby increasing strength, agility or speed beyond the normal human range. |
|  |  |

**Supplement Information 4:** Multivariate ordered logit regression models on the attitudes towards enhancement pills.^a^

|  |  |  |  |  |
| --- | --- | --- | --- | --- |
|  | **Model 1** | **Model 2** | **Model 3** | **Model 4** |
|  | **Item A** | **Item B** | **Item C** | **Item D** |
|  |  |  |  |  |
|  |  |  |  |  |
| Europ. country *(Ref.* | 0.56 | 1.63 | 1.43 | 0.94 |
| *Non-Europ. Country)* | [0.28;1.12] | [0.85;3.13] | [0.66;3.10] | [0.46;1.91] |
| Sea *(Ref. Land)* | 0.58* | 1.59 | 0.96 | 0.34*** |
|  | [0.36;0.92] | [0.98;2.59] | [0.53;1.74] | [0.20;0.57] |
| Air *(Ref. Land)* | 0.76 | 2.10** | 1.13 | 0.79 |
|  | [0.46;1.25] | [1.23;3.62] | [0.58;2.19] | [0.43;1.44] |
| Civil Service *(Ref. Land)* | 0.56 | 1.04 | − ^b^ | 0.85 |
|  | [0.15;2.11] | [0.22;4.96] |  | [0.20;3.66] |
| Medical branch *(Ref. not* | 0.97 | 0.72 | 0.71 | 0.39 |
| *in medical branch)* | [0.40;2.35] | [0.25;2.08] | [0.19;2.65] | [0.12;1.31] |
| Number of deployments | 1.00 | 0.96* | 0.98 | 1.03 |
|  | [0.97;1.04] | [0.92;1.00] | [0.94;1.02] | [0.99;1.08] |
| Cohort 2 *(Ref. Cohort 1)* | 1.01 | 1.12 | 1.08 | 0.74 |
|  | [0.54;1.91] | [0.59;2.12] | [0.51;2.28] | [0.36;1.50] |
| Cohort 3 *(Ref. Cohort 1)* | 0.56 | 1.63 | 1.43 | 0.94 |
|  | [0.28;1.12] | [0.85;3.13] | [0.66;3.10] | [0.46;1.91] |
| Cohort 4 *(Ref. Cohort 1)* | 0.58* | 1.59 | 0.96 | 0.34*** |
|  | [0.36;0.92] | [0.98;2.59] | [0.53;1.74] | [0.20;0.57] |
| Cohort 5 *(Ref. Cohort 1)* | 0.76 | 2.10** | 1.13 | 0.79 |
|  | [0.46;1.25] | [1.23;3.62] | [0.58;2.19] | [0.43;1.44] |
| Observations | 332 | 332 | 332 | 332 |
| *AIC* | 1246.3 | 950.34 | 565.54 | 824.21 |
| *BIC* | 1307.18 | 1011.22 | 622.62 | 885.09 |
|  |  |  |  |  |
|  |  |  |  |  |

*Notes:* **A** The military should supply pills that enhance performance beyond the normal range to support mission goals. **B** Individual military personnel should be allowed to refuse an order to take pills that enhance performance beyond the normal range in missions. **C** Individual military personnel should be allowed to freely decide about participation in military research on pills that enhance performance beyond the normal range. **D** The military should conduct research to test the safety and efficacy of pills that enhance performance beyond the normal range on military personnel who will be using them in the field.

**p*<.05, ***p*<.01, ****p*<.001; Adjusted odds ratios and 95% confidence intervals in brackets. ^a^ Wald post-estimation tests show that in none of the models a statistically significant difference occurred between Sea and Air, Sea and Civil Service, as well as Air and Civil Service and between cohort 2, 3, 4, and 5. The only exceptions are in Model 3 (here cohort 5 differs significantly from cohort 2 (*p*=.041) and 4 (*p*=.045)) and Model 4 (Sea and Air differ significantly, *p*=.005); ^b^ No estimation possible due to homogeneity of responses and low case-numbers.

**Supplement Information 5:** Pairwise correlations of the attitudes about enhancement pills (*N=*332).

|  | A | B | | C | | D | |  |
| --- | --- | --- | --- | --- | --- | --- | --- | --- |
|  |  |  | |  | |  | |  |
| A) The military should supply pills that enhance performance beyond the normal range to support mission goals | -1.00*** |  | |  | |  | |  |
|  |  |  | |  | |  | |  |
| B) Individual military personnel should be allowed to refuse an order to take pills that enhance performance beyond the normal range in missions | -0.24*** | -1.00*** | |  | |  | |  |
|  |  |  | |  | |  | |  |
| C) Individual military personnel should be allowed to freely decide about participation in military research on pills that enhance performance beyond the normal range. | -0.00*** | -0.22*** | | -1.00*** | |  | |  |
|  |  |  | |  | |  | |  |
| D) The military should conduct research to test the safety and efficacy of pills that enhance performance beyond the normal range on military personnel who will be using them in the field. | -0.39*** | -0.14*** | | -0.11*** | | -1.00*** | |  |
|  |  | |  | |  | |  | |

*Notes:* *N=*Number of observations; **p*<.05, ****p*<.001.

**Supplement Information 6:** Multivariate ordered logit regression models on the attitudes towards neural implants.^a^

|  | **Model 1** | **Model 2** | **Model 3** | **Model 4** | **Model 5** | **Model 6** |
| --- | --- | --- | --- | --- | --- | --- |
|  | **Item A** | **Item B** | **Item C** | **Item D** | **Item E** | **Item F** |
|  |  |  |  |  |  |  |
|  |  |  |  |  |  |  |
| Europ. country *(Ref.* | 1.58 | 2.38* | 0.73 | 0.76 | 0.34** | 0.71 |
| *Non-Europ. Country)* | [0.73.3.40] | [1.05;5.42] | [0.38;1.38] | [0.39;1.47] | [0.18;0.67] | [0.37;1.38] |
| Sea *(Ref. Land)* | 0.81 | 0.63 | 0.65 | 0.93 | 0.85 | 0.70 |
|  | [0.45;1.46] | [0.31;1.28] | [0.41;1.03] | [0.59;1.48] | [0.53;1.34] | [0.43;1.13] |
| Air *(Ref. Land)* | 1.19 | 0.74 | 0.9 | 0.99 | 0.99 | 0.83 |
|  | [0.61;2.33] | [0.35;1.57] | [0.55;1.48] | [0.60;1.61] | [0.60;1.63] | [0.49;1.39] |
| Civil Service *(Ref. Land)* | − ^b^ | 0.94 | 0.63 | 0.48 | 0.81 | 3.08 |
|  |  | [0.10;8.98] | [0.16;2.47] | [0.12;1.94] | [0.21;3.15] | [0.58;16.35] |
| Medical branch *(Ref. not* | 0.68 | 0.70 | 0.88 | 0.64 | 0.39 | 0.42 |
| *in medical branch)* | [0.18;2.53] | [0.15;3.31] | [0.36;2.17] | [0.26;1.59] | [0.15;1.01] | [0.15;1.17] |
| Number of deployments | 0.96* | 1.00 | 0.97 | 0.97 | 0.99 | 1.02 |
|  | [0.92;1.00] | [0.95;1.05] | [0.94;1.01] | [0.93;1.00] | [0.96;1.03] | [0.98;1.06] |
| Cohort 2 *(Ref. Cohort 1)* | 1.14 | 1.00 | 0.57 | 0.65 | 0.80 | 1.01 |
|  | [0.53;2.45] | [0.41;2.42] | [0.30;1.08] | [0.35;1.21] | [0.43;1.50] | [0.53;1.93] |
| Cohort 3 *(Ref. Cohort 1)* | 1.58 | 2.38* | 0.73 | 0.76 | 0.34** | 0.71 |
|  | [0.73.3.40] | [1.05;5.42] | [0.38;1.38] | [0.39;1.47] | [0.18;0.67] | [0.37;1.38] |
| Cohort 4 *(Ref. Cohort 1)* | 0.81 | 0.63 | 0.65 | 0.93 | 0.85 | 0.70 |
|  | [0.45;1.46] | [0.31;1.28] | [0.41;1.03] | [0.59;1.48] | [0.53;1.34] | [0.43;1.13] |
| Cohort 5 *(Ref. Cohort 1)* | 1.19 | 0.74 | 0.90 | 0.99 | 0.99 | 0.83 |
|  | [0.61;2.33] | [0.35;1.57] | [0.55;1.48] | [0.60;1.61] | [0.60;1.63] | [0.49;1.39] |
| Observations | 332 | 332 | 332 | 332 | 332 | 332 |
| *AIC* | 567.45 | 412.67 | 1239.17 | 1303.75 | 1239.88 | 944.96 |
| *BIC* | 620.72 | 465.94 | 1300.05 | 1364.63 | 1300.76 | 1005.84 |

*Notes:* **A** Individual military personnel should be allowed to freely decide about participation in military research on neural implants that enhance functioning beyond the normal range. **B** Prior to surgery, military personnel should be informed whether or not they can keep a military-supplied neural implant that enhances functioning, after they leave the military. **C** The military should base decisions about whether or not to remove neural implants from military personnel on whether the implant primarily treats a condition (e.g., a cochlear implant to treat hearing loss) or whether it enhances function beyond the normal range (e.g., a cochlear implant that provides 20-times the hearing power of a normal person). **D** A neural implant provided to military personnel during military service to enhance functioning beyond the normal range is the property of the military. **E** It is dangerous for society if a soldier were to keep a neural implant from the military that enhances functioning, after leaving the military. **F** Veterans who have lost eyesight or hearing as a result of military missions should be prioritized for neural implants that allow normal functioning when these reach the civilian market.

**p*<.05, ***p*<.01, ****p*<.001; Adjusted odds ratios and 95% confidence intervals in brackets. a Wald post-estimation tests show that in none of the models a statistically significant difference occurred between Sea and Air, Sea and Civil Service, as well as Air and Civil Service and between cohort 2, 3, and 4. The only exceptions are in Model 1 (here cohort 4 differs significantly from cohort 3 (*p*=.026) and 5 (*p*=.037)); ^b^ No estimation possible due to homogeneity of responses and low case-numbers.

**Supplement Information 7:** Pairwise correlations of the attitudes about neural implants (*N=*332).

|  | A | B | C | D | E | F |
| --- | --- | --- | --- | --- | --- | --- |
|  |  |  |  |  |  |  |
| A) Individual military personnel should be allowed to freely decide about participation in military research on neural implants that enhance functioning beyond the normal range. | -1.00*** |  |  |  |  |  |
|  |  |  |  |  |  |  |
| B) Prior to surgery, military personnel should be informed whether or not they can keep a military-supplied neural implant that enhances functioning, after they leave the military. | -0.27*** | -1.00*** |  |  |  |  |
|  |  |  |  |  |  |  |
| C) The military should base decisions about whether or not to remove neural implants from military personnel on whether the implant primarily treats a condition (e.g., a cochlear implant to treat hearing loss) or whether it enhances function beyond the normal range (e.g., a cochlear implant that provides 20-times the hearing power of a normal person). | -0.06*** | -0.05*** | -1.00*** |  |  |  |
|  |  |  |  |  |  |  |
| D) A neural implant provided to military personnel during military service to enhance functioning beyond the normal range is the property of the military. | -0.05*** | -0.08*** | -0.47*** | -1.00*** |  |  |
|  |  |  |  |  |  |  |
| E) It is dangerous for society if a soldier were to keep a neural implant from the military that enhances functioning, after leaving the military. | -0.02*** | -0.07*** | -0.42*** | -0.51*** | -1.00*** |  |
|  |  |  |  |  |  |  |
| F) Veterans who have lost eyesight or hearing as a result of military missions should be prioritized for neural implants that allow normal functioning when these reach the civilian market. | -0.14*** | -0.20*** | 0.01*** | -0.11*** | 0.01*** | -1.00*** |
|  |  |  |  |  |  |  |

*Notes:* *N=*Number of observations; ***p*<.01, ****p*<.001.

**Supplement Information 8:** Multivariate ordered logit regression models on the attitudes towards neuroprostheses.^a^

|  | **Model 1** | **Model 2** | **Model 3** | **Model 4** | **Model 5** | **Model 6** |
| --- | --- | --- | --- | --- | --- | --- |
|  | **Item A** | **Item B** | **Item C** | **Item D** | **Item E** | **Item F** |
|  |  |  |  |  |  |  |
|  |  |  |  |  |  |  |
| Europ. country *(Ref.* | 1.7 | 1.67 | 0.87 | 0.76 | 0.66 | 0.92 |
| *Non-Europ. Country)* | [0.77;3.78] | [0.73;3.83] | [0.46;1.63] | [0.39;1.47] | [0.34;1.27] | [0.48;1.76] |
| Sea *(Ref. Land)* | 1.11 | 0.85 | 0.79 | 0.93 | 1.05 | 0.62 |
|  | [0.61;2.02] | [0.44;1.64] | [0.50;1.26] | [0.59;1.48] | [0.66;1.67] | [0.38;1.02] |
| Air *(Ref. Land)* | 1.57 | 0.93 | 0.84 | 0.99 | 0.9 | 0.77 |
|  | [0.79;3.11] | [0.46;1.88] | [0.51;1.38] | [0.60;1.61] | [0.55;1.48] | [0.46;1.29] |
| Civil Service *(Ref. Land)* | − ^b^ | 1.27 | 0.69 | 0.48 | 0.91 | 6.13 |
|  |  | [0.14;11.79] | [0.16;2.98] | [0.12;1.94] | [0.23;3.55] | [0.72;52.29] |
| Medical branch *(Ref. not* | 0.68 | 0.58 | 0.56 | 0.64 | 0.60 | 0.49 |
| *in medical branch)* | [0.18;2.53] | [0.12;2.72] | [0.22;1.44] | [0.26;1.59] | [0.22;1.59] | [0.17;1.36] |
| Number of deployments | 0.94** | 1.01 | 0.99 | 0.97 | 0.98 | 1.01 |
|  | [0.90;0.98] | [0.96;1.06] | [0.95;1.02] | [0.93;1.00] | [0.95;1.02] | [0.98;1.05] |
| Cohort 2 *(Ref. Cohort 1)* | 3.18** | 2.44* | 1.00 | 0.8.0 | 1.05 | 1.39 |
|  | [1.48;6.85] | [1.06;5.62] | [0.56;1.81] | [0.45;1.43] | [0.59;1.88] | [0.75;2.58] |
| Cohort 3 *(Ref. Cohort 1)* | 1.85 | 1.74 | 1.04 | 0.71 | 0.77 | 1.38 |
|  | [0.92;3.73] | [0.79;3.83] | [0.58;1.88] | [0.40;1.25] | [0.43;1.37] | [0.74;2.56] |
| Cohort 4 *(Ref. Cohort 1)* | 3.74** | 1.56 | 0.96 | 1.20 | 0.75 | 0.74 |
|  | [1.46;9.55] | [0.65;3.74] | [0.49;1.85] | [0.62;2.33] | [0.38;1.47] | [0.37;1.47] |
| Cohort 5 *(Ref. Cohort 1)* | 1.65 | 1.65 | 0.64 | 0.65 | 1.03 | 1.02 |
|  | [0.78;3.49] | [0.71;3.84] | [0.34;1.18] | [0.35;1.21] | [0.55;1.93] | [0.53;1.97] |
| Observations | 332 | 332 | 332 | 332 | 332 | 332 |
| *AIC* | 555.34 | 428.04 | 1223.9 | 1303.75 | 1251.27 | 910.08 |
| *BIC* | 612.42 | 477.51 | 1284.79 | 1364.63 | 1312.15 | 970.96 |

*Notes:* **A** Individual military personnel should be allowed to freely decide about participation in military research on neuroprostheses that enhance functioning beyond the normal range. **B** Prior to surgery, military personnel should be informed whether or not they can keep a military-supplied neuroprosthesis that enhances functioning, after they leave the military. **C** The military should base decisions about whether or not to remove neuroprostheses from military personnel on whether the prostheses primarily treats a condition (e.g., a mind-controlled prosthesis for a lost hand to enable gripping) or whether it enhances function beyond the normal range (e.g., a mind-controlled prosthesis that provides 20-times the grip power of a normal hand). **D** A neuroprosthesis provided to military personnel during military service to enhance functioning beyond the normal range is the property of the military. **E** It is dangerous for society if a soldier were to keep a neuroprosthesis from the military that enhances functioning, after leaving the military. **F** Veterans who have lost limbs as a result of military missions should be prioritized for neuroprostheses that allow normal functioning when these reach the civilian market.

**p*<.05, ***p*<.01, ****p*<.001; Adjusted odds ratios and 95% confidence intervals in brackets. ^a^ Wald post-estimation tests show that in none of the models a statistically significant difference occurred between Sea and Air, Sea and Civil Service, as well as Air and Civil Service and between cohort 2, 3, and 4. The only exception is in Model 6 (Air and Civil Service differ significantly, *p*=.038); ^b^ No estimation possible due to homogeneity of responses and low case-numbers.

**Supplement Information 9:** Pairwise correlations of the attitudes about neuroprostheses (*N=*332).

|  | A | B | C | D | E | F |
| --- | --- | --- | --- | --- | --- | --- |
|  |  |  |  |  |  |  |
| A) Individual military personnel should be allowed to freely decide about participation in military research on neuroprostheses that enhance functioning beyond the normal range. | -1.00*** |  |  |  |  |  |
|  |  |  |  |  |  |  |
| B) Prior to surgery, military personnel should be informed whether or not they can keep a military-supplied neuroprosthesis that enhances functioning, after they leave the military. | -0.31*** | -1.00*** |  |  |  |  |
|  |  |  |  |  |  |  |
| C) The military should base decisions about whether or not to remove neuroprostheses from military personnel on whether the prostheses primarily treats a condition (e.g., a mind-controlled prosthesis for a lost hand to enable gripping) or whether it enhances function beyond the normal range (e.g., a mind-controlled prosthesis that provides 20-times the grip power of a normal hand). | -0.09*** | -0.00*** | -1.00*** |  |  |  |
|  |  |  |  |  |  |  |
| D) A neuroprosthesis provided to military personnel during military service to enhance functioning beyond the normal range is the property of the military. | -0.00*** | -0.06*** | -0.50*** | -1.00*** |  |  |
|  |  |  |  |  |  |  |
| E) It is dangerous for society if a soldier were to keep a neuroprosthesis from the military that enhances functioning, after leaving the military. | -0.03*** | -0.07*** | -0.47*** | -0.55*** | -1.00*** |  |
|  |  |  |  |  |  |  |
| F) Veterans who have lost limbs as a result of military missions should be prioritized for neuroprostheses that allow normal functioning when these reach the civilian market. | -0.21*** | -0.18*** | -0.10*** | -0.10*** | -0.02*** | -1.00*** |
|  |  |  |  |  |  |  |

*Notes:* *N=*Number of observations; ****p*<.001.
